# A Framework for Detecting and Analyzing Behavior Changes of Elderly People over Time Using Learning Techniques

**DOI:** 10.3390/s20247112

**Published:** 2020-12-11

**Authors:** Dorsaf Zekri, Thierry Delot, Marie Thilliez, Sylvain Lecomte, Mikael Desertot

**Affiliations:** 1 LAMIH UMR CNRS 8201, Université Polytechnique Hauts-de-France, 59300 Valenciennes, France; thierry.delot@uphf.fr (T.D.); Marie.Thilliez@uphf.fr (M.T.); Mikael.Desertot@uphf.fr (M.D.); 2 ReDCAD Laboratory, University of Sfax, B.P. 1173, 3029 Sfax, Tunisia; 3 IMT Lille Douai, Digital Systems Center, Institut Mines-Telecom, University of Lille, 59000 Lille, France; sylvain.lecomte@imt-lille-douai.fr

**Keywords:** behavior change observation, elderly people, smart home, activities of daily living, decision support system, fuzzy logic system

## Abstract

A sensor-rich environment can be exploited for elder healthcare applications. In this work, our objective was to conduct a continuous and long-term analysis of elderly’s behavior for detecting changes. We indeed did not study snapshots of the behavior but, rather, analyzed the overall behavior evolution over long periods of time in order to detect anomalies. Therefore, we proposed a learning method and formalize a normal behavior pattern for elderly people related to her/his Activities of Daily Living (ADL). We also defined a temporal similarity score between activities that allows detecting behavior changes over time. During the periods of time when behavior changes occurred, we then focused on each activity to identify anomalies. Finally, when a behavior change occurred, it was also necessary to help caregivers and/or family members understand the possible pathology detected in order for them to react accordingly. Therefore, the framework presented in this article includes a fuzzy logic-based decision support system that provides information about the suspected disease and its severity.

## 1. Introduction

With the growing elderly population [1,2], numerous research works have focused on preserving independent living of elderly people. Elderly people often suffer from several interacting problems [3,4], due to loss of function or difficulties in interacting with their environment. All these factors, separately or together, obviously determine the elderly person’s level of independence and influence his/her quality of life.

In this context, most researchers aim to improve the living conditions of elderly people with respect to medical issues, such as diabetes or cognitive disabilities, by analyzing the behavior of residents within sensor-based environments [5]. The progress of technology (wearable sensors, smartphones and other mobile devices, wireless communications, etc.) indeed enables the development of effective solutions to help elderly people continue living independently in their smart homes [6,7,8,9].

The smart home concept consists of houses equipped with simple environmental sensors and more complex systems including audio, video, or bio-metric systems. The raw information captured by the sensors can obviously not be shared as such with the medical staff or used directly to automatically detect changes in behavior. However, knowledge extracted from these data can be used to enrich the information delivered to the medical staff and improve the precision of an early detection. There is evidence that opportunistic home surveillance prevents in some cases hospitalization.

An at-home monitoring of elderly person’s behavior may help to detect autonomy loss and sudden changes of the health status [10,11,12]. In the case of elderly people, a change in the behavior usually indicates a disturbance in their health status or independence level, especially if this change impacts daily activities and lasts over time. Contrary to a sudden problem, like the fall of a person [13], it is non-trivial to detect, at the early stage, a pathology that will develop over a long period of time, like a cognitive disease, even using sensor data. Hence, the identification of abnormal patterns, especially in daily activities monitoring systems over a long period of time, can be valuable for caregivers and/or family members. This may indeed enable the detection of a silent disease as soon as possible, and thus allow them to make the right decisions to actually assist elderly people.

In this article, we address the problem of monitoring the behavior evolution using processed and annotated sensor data, collected in a smart home context, where elderly person’s activities were identified. The originality of this work is in proposing a framework to conduct a continuous and long-term analysis of elderly person’s behavior for detecting changes. We indeed did not study snapshots of the behavior but analyzed the overall behavior evolution over long periods of time in order to detect anomalies (e.g., the elderly starts sleeping less and less). Finally, once an anomaly in the behavior is detected, our solution analyzes the situation to actually help caregivers assist elderly people.

Our contribution in this article consists in designing a framework for automatically detecting behavior changes over a long period of time. This framework includes decision-making features based on fuzzy logic to assist family members or caregivers in the disease assessment phase.

The rest of this paper is organized as follows. In Section 2, we discuss about different research works related to our study. Section 3 details our approach to monitor the behavior of elderly people, detect anomalies and identify possible underlying diseases. In Section 5, an experimental study is proposed to validate our approach. In Section 4, we report the experimentation of our proposal on real datasets. Finally, we give our conlusions and introduce future works in Section 6.

## 2. Related Works

Numerous recent projects and applications achieved in the area of Smart Homes have focused on improving access to healthcare services and enhancing assisted living for elderly people. In the following, we first introduce the most recent smart home projects for elderly healthcare services and then present several research works focusing on behavior change detection for elderly people.

### 2.1. Smart Home Projects for Elderly Healthcare Services

The CASAS (Center for Advanced Studies in Adaptive Systems) smart home project (http://casas.wsu.edu/) [14], developed at Washington State University, is a multi-disciplinary research project focused on creating an intelligent home environment by using unobtrusive sensors and actuators. The research areas covered by the CASAS project include assistive technology, artificial intelligence, machine learning, and activity recognition. The benchmark dataset described in reference [14] was also made available as part of this project and is widely used to analyze behavior of all types of people including elderly people [15,16]. One of the works developed in the CASAS project concerns the design of a lightweight smart home called “smart home in a box” [17]. This work uses unsupervised learning for activity recognition and activity discovery.

The SWEET-HOME project (http://sweet-home.imag.fr/index.php?choix=projet) [18] focuses on the design of a new smart home system based on audio technology. This audio-based interaction technology is used to control the home environment and help elderly people to easily live their social life. An interesting research direction of the SWEET-HOME project concerns the context-aware decision process [19], which uses Markov Logic Networks to facilitate the detection of uncertain events inferred from real sensor data.

A third project which aims to provide health-care assistance in smart home environments is the Unobtrusive Smart Environments for Independent Living (USEFIL) project (https://www.usefil.eu/) [20]. In this project, a set of sensors and devices (e.g., a wrist camera and a Kinect sensor) are used to identify the basic physical activities of elderly people, such as walking, etc. [21].

The E-Health Monitoring Open Data Project (http://www.ehealthmonitor.eu/index.php?content=home) [22,23] provides free online datasets related to the monitoring of dependent people, such as elderly people. Data are generated using simulations or collected from real life platforms using real sensors. Targeted systems are typically smart home-health (or institution-health) monitoring systems. This project also provides a large amount of public datasets researchers can use to conduct further studies in testing and validating innovative E-health services and solutions. In this project, a decision-making system is proposed for patient-physician relationships using a multi-agent organization. The resulting software system captures all relevant patient-physician relationships, roles, and permissions and allows for individualized decision support in distributed knowledge sharing environments [24].

### 2.2. ADL Detection in Smart Environments

Recognizing the activities of daily living (ADL) for people living in smart homes is the basis of unobtrusive wellness monitoring. ADLs is a general term used by healthcare professionals to refer to the basic self-care tasks an individual does on a day-to-day basis. These fundamental activities are obviously crucial for evaluating or maintaining independence. They are indeed used by healthcare professionals as a way to measure an individual’s functional status, especially for elderly people [25]. Several research works also exploit ADLs as the building block to construct applications, such as healthcare monitoring or ambient assisted living [26].

In the context of smart homes, the daily activities of residents can be detected through sensors embedded within various areas in the home. In the literature, various methods have been proposed [27,28,29,30,31,32] to detect activities of daily life for elderly people at home using passive infrared (PIR) motion sensors, body-worn sensors, pressure sensors, video monitoring, or sound recognition. Bouchard et al. [33] describe their work on human activities recognition in smart homes using passive RFID (Radio Frequency IDentification) tags. They aim to identify human activities with a minimal rate of false positives. In the same context, Reference [7] presents an approach to recognize human activities using body postures estimated from RGB-D (RedGreenBlue-Depth ) data. This work focuses on recognizing complex activities composed of sequential or simultaneous atomic actions characterized by body motions of a single actor.

The output of the works previously cited in this section consists in a list of human activities including Activities of Daily Living (ADLs). In Reference [34,35], Katz et al. subdivided ADLs in two categories:

1. Basic ADL (BADLs) are self-care activities routinely performed which include, but are not limited to, five activities: sleeping, dressing, eating (three meals), and going to toilet, hygiene activities (e.g., take a shower).
2. Instrumental ADL (IADLs) [36] are domestic activities that are not strictly necessary (i.e., secondary activities) but allow a person to live independently. This type of activities includes going outside, household shopping, preparing food, taking drugs, and using the phone.

ADLs can be qualified according to several criteria [37]: time of the day, duration of the activity, location in the house, and sequence of actions.

### 2.3. Anomalies Detection in Elderly Behavior

Behavior anomaly detection refers to the problem of finding unusual or abnormal patterns (that do not conform to expected behavior) in the data describing the user’s behavior. For example, a person lying down on the floor for a long time without any movement is an unusual behavior. This topic is highly relevant in the context of ADL and sensor data in smart homes and should be considered as critical for the deployment of elderly surveillance systems.

There are fundamentally three different strategies to analyze the behavior for elderly people in the context of smart homes and detect anomalous behaviors: (1) the activities recognition strategy (2) the discriminating and profiling strategies. In the following sections, we first introduce the different types of anomaly and then detail these strategies.

#### 2.3.1. Activity Recognition for Anomalous Behavior Detection

In the reviewed literature, various methods have been proposed to detect an abnormal behavior using daily activities [11,12,38,39,40,41] in a spatio-temporal context using different techniques/methods (classification, clustering, nearest neighbor, statistical, etc.). Such systems implicitly rely on the recognition and the representation of human activities. These approaches are grouped, as in reference [39,42,43], into three different classes, namely point anomaly, collective anomaly, and contextual anomaly

1. Point anomaly considers each activity independently and decides whether it is normal or not, with respect to the normal behavior. To do so, Reference [44,45] studies elderly residents diagnosed with dementia living independently in real home environments. Neural networks and clustering algorithms are used by these works to predict sensor activity. In the same context, Sprint et al. [46] use sensor data for the activity "to sleep" as inputs to change detection algorithms, such as RuLSIF (Relative unconstrained Least-Squares Importance Fitting), virtual classifier, and sw-PCAR (small window adaptation of the Permutation-based Change Detection in Activity Routine algorithm), to detect and analyze behavior changes that accompany health events. In Reference [47,48,49], the authors propose solutions to detect anomalies related to mild cognitive impairment and represent them as propositional logic. According to rules defined by experts, the anomaly is detected as an activity containing a deviation with respect to the normal behavior.
2. Collective anomaly exploits groups of activity instances together to determine whether the group is normal or not. For instance, Gjoreski et al. [50] have proposed a system to monitor users’ daily activity by combining accelerometers with an electrocardiogram (ECG) sensor. Measured acceleration data can thus be analyzed in conjunction with the ECG signals to detect anomalies in the user’s behavior and heart-related problems. Still in the health field, another work which considers correlations among several activities is presented in Reference [51]. This system applies anomaly detection on wearable sensors to provide an intelligent living environment for elderly residents. The detection of anomalies is based on several parameters: location, time, duration, type of activity, and transitions between activities. The experiments provided consist of a semi-supervised learning approach.
3. Contextual anomaly considers activities under a particular context (e.g., day of week, person under medication, etc.). For instance, in Reference [40], the authors propose a technique to detect contextually anomalous values in streaming sensor systems. In this work, anomalies have a dimensional and contextual locality identified using a contextual anomaly detection algorithm with contextual clustering. In the same context, Zhu et al. [52] proposed a system for detecting anomalous activities based on context aware activity recognition based on video. This system includes a structural model to learn the context and motion patterns from training sets of activities.

#### 2.3.2. Discriminating and Profiling for Anomalous Behavior Detection

Once the sensor data is well recognized and annotated, anomalies in the human behavior can be detected by examining activity patterns which do not match or deviate with respect to the normal behavior pattern. The basic way to identify anomalies is to look at the data points that do not conform to the properties of the regular pattern. Two different strategies have, therefore, been introduced in the literature [10,43]: discriminating and profiling which is based on behavior modeling [53,54,55] to build a normal behavior.

1. With the discriminating strategy, anomaly data are defined according to previously collected/ historical anomalous data. This strategy assumes that abnormal events already occurred before (in the training set). The incoming data is so matched against the previously recorded behavioral pattern to detect a possible anomaly. The approach presented in Reference [56] falls into this category. In this work, the authors propose a semi-supervised adapted Hidden Markov Model (HMM) framework, in which usual event models are first learned from a training data, while unusual event models are learned by Bayesian adaptation in an unsupervised manner. In another domain, an approach for recognizing rare events in aerial video is proposed in Reference [57]. This approach relies on Hidden Markov Models (HMMs) to represent the spatio-temporal relationships between objects, as well as the uncertainty in observations.
  While it might be possible to build discriminative models of abnormality [56,57], abnormal behaviors can occur in countless forms in other applications; therefore, it appears problematic to be able to build a good generic model of abnormality. It is also unrealistic to obtain a large training data set for unusual events, especially in a person’s daily life context.
2. With the profiling strategy, a model of normality is built and the new incoming or observed data is compared with the model. It is then possible to detect any deviation from this model. In such a case, the behavior is considered as an anomaly if it deviates or does not match with the normal model. The first important step with this strategy resides in the construction of the normal behavior. A Bayesian formulation is provided for profiling and behavioral anomaly detection in Reference [44] and applied to elderly people who live alone at home. The normal behavior of the residents are extracted using Bayesian statistics, based on the raw measures of users activity with event sequence and event duration. Many approaches use clustering to build the behavioral profile needed for detecting anomalous behaviors. For instance, the approach presented in Reference [58] proposes a probabilistic spatio-temporal model to summarize daily behavior by applying clustering (k-means algorithm) on the behavioral profile. Anomalies are then defined as significant changes from the learned behavioral model and detected using a cross-entropy measure. K-means clustering is also applied to separate the normal routine from unusual and suspected routines in Reference [59,60], where the authors recognize the pre-segmented activities of daily living using Probabilistic Neural Network (PNN). The anomalies are categorized based on criteria, such as missing or extra subevents or an unusual duration of the activity. In Reference [61], the authors proposed a new approach to monitor activities dedicated to older adults. In this approach, the behavior routine is given by the older adult. An experimenter, asks her/him to sketch each activity. The outcome is a pattern of daily activities. Using these patterns, the authors propose a score to evaluate how strictly an activity matches with the user’s routine. This activity score is comprised between 0 and 1. Zero means that the activity has not been performed, according to the user’s routine. The value 1 indicates that the sensed measures strictly match with the user’s routine. In Reference [61], the approach is applied to five ADLs (meals preparation, go to bed, get up, taking a shower, and getting dressed). Each activity has a specific score formula, taking into account the occurrence time but not the activity duration.

### 2.4. Discussion

Previous research works focus on the analysis of the behavior to detect possible anomalies in the elderly person’s behavior. Several works rely on the recognition of the human activity to detect anomalies, such as Reference [50,51], which exploit wearable sensors to monitor vital signs, whereas Reference [10,11,12,44,45,46] considers home sensors to monitor daily activities. However, these works do not analyze all the activities of the elderly person at the same time. For example, the work presented in Reference [10] focuses on the behavior changes related to sleeping only. Moreover, all the solutions mentioned previously have been developed with the objective to quickly detect and react when a sudden behavior change occurs. The typical example of such an event is “the fall” of the monitored person [13].

In this article, we do not focus on the recognition phase of the human activities. We rather assume that this phase has already been performed and consider available datasets containing the detail of identified activities.

Besides, our objective is not to detect sudden changes in the behavior, like the fall of a person, but rather to monitor and analyze the evolution of the behavior over a long period of time using previously labeled activities. Thus, we can detect a slow and silent evolution in the elderly person’s routine in order to notify caregivers and/or family members. Moreover, a decision-making system included in our framework can assist them to identify the observed pathology and thus take the appropriate decision. Our proposal belongs to the category of works based on the construction normal behavior patterns, and so follows the profiling strategy introduced in Section 2.3.2. Most of the approaches ranked in this category [58,59,60] use the K-means clustering algorithm to build the normal behavior pattern. This algorithm requires as input the number of clusters which should be identified. Among the works using normal behavior patterns, the solution proposed by Caroux et al. [61] allows the detection of long-term behavior change using activities scores. It, therefore, shares the same objective as our framework in this article. This work, however, does not consider ADLs, like toileting and leaving home. However, studying these two activities can give an idea on both the health situation and the level of autonomy of the elderly. Indeed, if the elderly starts leaving home less frequently, as well as going to toilet more or less than usual, this may mean that the elderly person is losing her/his autonomy over time or is suffering from an illness. Moreover, Caroux et al. [61] do not include the activity duration which is yet considered among the key elements to detect the loss of autonomy [23,41]. For example, if the elderly person sleeps less and less every month, this can be worrying and may mean that the person is suffering from a disease.

## 3. Our Framework for Detecting and Analyzing Behavior Changes

The overall objective of this study is to analyze the evolution of the daily behavior of elderly people living in their apartment over a long period of time using processed and annotated ambient sensor data that has been identified for elderly’s activities. The results obtained may indeed be useful to help the family members and/or the caregivers to assist elderly people in the best way and make appropriate decisions according to behavior changes. In Section 3.1, we first present how the behavior is modeled and then explain how anomalies are detected. In the following one (Section 3.2), we introduce our decision support system for caregivers and family members based on the person’s behavior, profile and routine.

### 3.1. Detection of Elderly Person’s Behavior Change Over Time

In the following, we introduce our model to characterize the normal behavior pattern for elderly people, which can then be used to detect behavior changes over time by comparing the current behavioral data of an elderly with her/his usual behavior pattern.

#### 3.1.1. Activities and Daily Behavior Pattern

In the scope of this article, we consider several monitored ADLs, related to ADL categories explained in Section 2.2, for detecting anomalies, and more precisely:

- 4 Basic ADL activities: sleeping, eating (breakfast, lunch, dinner), taking a shower, and going to toilet.
- 1 Instrumental ADL: leaving home.

In the following, each activity is associated with two key criteria, also used in reference [62]:

- Location: the specific place where an activity occurs. For example, the eating activity may take place in the kitchen.
- Time: the duration and occurring time associated to an activity. The monitored person may perform a same activity at different times of the day (e.g., going to toilet), whereas some other activities only occur at specific times of the day (e.g., the activity eating breakfast should occur in the morning (between 6 a.m. and 11 a.m.) and should last between 15 and 45 min). In the following, we assume that the start time and duration of each activity instance is detected by an activity recognition system based on in-home sensors.

In our work, activities are a semantic interpretation of sensor measurements. They represent the building block of the daily behavior pattern of the elderly person, describing how the user performs her/his activities at different times and models relationships between them.

Let A={$a_{1};a_{2};...;a_{4}$} be the set of activities labels. An activity pattern represents when and where an activity usually occurs. It is defined as a tuple:

$$P_{a}=\left(a_{i},S_{a}\left(t\right),D_{a}\left(t\right)\right),$$

where:

- $a_{i}\in A$ is an activity label.
- $S_{a}\left(t\right)$ is a time interval representing the usual start time of activity $a_{i}$.
- $D_{a}\left(t\right)$ is a time interval representing the usual duration of activity $a_{i}$.

The daily behavior pattern involves a sequence of activity patterns. It defines order constraints on them and introduces possible temporal delays. It is built from data derived from the sensors deployed in a smart home.

$$B=(P_{a1},P_{a2},P_{a3})，\;\mathrm{where}\;P_{ai}\;\mathrm{is}\;\mathrm{an}\;\mathrm{activity}\;\mathrm{pattern}.$$

For each day *i* of the week $D_{i}$, we built a behavior pattern $B_{i}$ which is a set of segments $P_{ai}$, where each segment $P_{ai}$ is a sequence of tuples ($a_{i},S_{a}\left(t\right),D_{a}\left(t\right)$) related to each activity. In this pattern, we include four activities: sleeping, eating, taking a shower and leaving home which occur at specific times of the day. The activity leaving home is sensitive to both the age-related functional decline and the elderly routine. Its regularity depends on both the health situation and the level of autonomy of the elderly people. In our work, the activity leaving home is only considered for elderly people who do not always stay at home (due to their health status for instance). The activity going to toilet may occur at many times during the day. It will be studied separately, as we will see later.

#### 3.1.2. Normal Behavior Pattern for the Elderly Person

The first step of any behavior anomaly detection system is to characterize the normal behavior of the person, also called routine behavior or regular behavior. This can be achieved using historical data about the person behavior to capture regularities in every individual activity. The normal behavior consists of the list of activities that a resident performs in her/his house, with the time of the day and duration. Thus, it captures the repetitive daily routines and deviations from the normal behavior may indicate changes of lifestyle or loss of capacity.

To build the routine behavior model, we follow several steps:

1. We first use data collected during the previous period which was treated as a baseline behavior period. In our experiments (see Section 4), an important part of the dataset will be dedicated to this step. In real conditions, a specified time frame (e.g., several weeks) should be used for characterizing the normal behavior. On these data, we then exploit unsupervised learning and apply clustering techniques to find point anomalies. To address this, we cluster instances of each activity based on start time and duration without considering the day of the week. For clustering, we use DBSCAN (Density-Based Spatial Clustering of Applications with Noise) [63], which is a density-based clustering algorithm. The major advantage of DBSCAN, compared with other clustering algorithms, like K-means, is that we do not need to specify how many clusters should be identified. DBSCAN marks each point as belonging to a cluster or as noise (anomaly).
2. Once the clustering step has been performed, we eliminate noise and compute the average start time and duration for each considered activity.

The activity going to toilet, that occurs several times a day, is also treated separately. The daily frequency is indeed usually regular for this activity. Moreover, it is not essential to be very precise on the start times here. Hence, we chose to focus on the frequency rather than the occurring time. In the following, we so consider the frequency, per n hours, for the activity going to toilet.

#### 3.1.3. Detection of Behavior Change Using a Daily Activity Score

Once computed, the normal behavior pattern can be used to detect anomalies by comparing the current behavioral data of an elderly with her/his normal behavior pattern. The basic idea of our behavioral deviation detection system is to estimate the similarity between both patterns using a score. We, therefore, consider three criteria for the activities: the time, duration and chronological order of the activities in the sequence.

Intuitively, a particular activity is similar to a pattern if its start time, duration and location are similar to the ones defined by the pattern. The similarity of the time and duration for each activity is estimated by a score.

The similarity score of an activity *a* occurring at day *d* with respect to the same activity in the normal behavior pattern $a_{n}$ is calculated by the Formula (1). It is given as a percentage and represents the temporal intersection of the normal behavior pattern and one observed day pattern, for the same activity. We note that $S_{ad}$ is the start time of activity $a_{d}$, $D_{ad}$ is the duration of activity $a_{d}$, and $E_{ad}=S_{ad}+D_{ad}$ is the end time of activity $a_{d}$.

(1) $$Similarity\;score=\frac{(\inf\left(E_{an},E_{ad}\right)-\sup\left(S_{an},S_{ad}\right))*100}{D_{an}}.$$

The similarity score for one day is the average of similarity scores for all activities occurring in this day.

The duration score is calculated by the Formula (2). It corresponds to the percentage of the duration of an activity in an observed day compared to the duration of the same activity in the normal behavior pattern.

(2) $$Duration\;score=\frac{D_{ad}*100}{D_{an}}.$$

The duration score for an activity on a particular day is the average of duration scores for all the instances of this activity occurring on this day. The duration score can obviously exceed 100% if the duration of an activity at the observed day is greater than the expected duration in the normal behavior pattern. This simply means that the elderly takes longer to achieve the activity, which may be caused by a loss of autonomy, especially if this phenomena increases over time.

**Figure 1:**
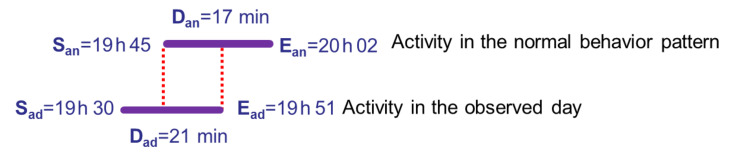
Illustration of the similarity and duration scores for the activity eating lunch.

In the example presented in Figure 1, the similarity score is 35.29%. It represents the temporal intersection that is the interval between both red dashed lines in Figure 1, for the activity eating lunch for one observed day compared to the same activity in the normal behavior pattern. The duration score (123.52% in our example) is the percentage of the duration of the activity eating lunch in an observed day compared to the duration of the same activity in the normal behavior pattern.

The variation of these scores over time represents the evolution of the elderly person’s life pace. These scores thus give us an indication of the variation with an elderly person’s usual behavior for a particular activity. A large decrease in these scores over a long period (from a few days to a few weeks) may be an initial signal of decline and should generate a notification to the caregivers or the family members.

For regular activities occurring several times a day and irregular activities occurring a few times in a week, we compare the frequency between a routine day and an observed day. By simply plotting the daily score along time, it is possible to identify certain days with unusual activities (i.e., with lower scores), or evolution trends that indicate deviations from the previous activity routine.

In this section, we described our solution to detect behavior changes for elderly people. Obviously, the scores produced may only be easily understood and exploited by experts, whereas our main objectives is to notify family members and caregivers about a possible pathology, without any prior knowledge about how our system works. Therefore, we propose in the following a decision support system to actually help family members and caregivers by identifying suspected diseases, thanks to the deviations observed in the normal behavior patterns.

### 3.2. Decision Support System

For elderly people, a change in the behavior may be the consequence of an appearing pathology. In the following, we so try to establish correlations between changes in the ADLs and diseases. Our goal here is to try to identify diseases in the elderly person as soon as their first symptoms appear.

Obviously, changes in the ADLs for an elderly person can match with several different diseases at the same time. For example, if an elderly person skips several meals, does not leave home for many days, and sleeps less and less, s/he may start suffering from a psychological disease (Alzheimer, stress, etc.). In our solution, we rely on the expertise of healthcare professionals to configure our decision support system and, more precisely, to define the diseases suspected and ADL that can be impacted by these diseases. Our system can be applied to each suspected disease to evaluate the disease level over time.

In the following, we focus on the design of our decision support system allowing caregivers and/or family members to take the right decision at the right time. Our decision support system is based on the person’s behavior. It exploits the scores introduced in Section 3.1.3 to effectively assist elderly people and uses fuzzy logic [64] in the suspected disease identification phase. It follows a two-step process. In the first step, we define the activities impacted by the suspected diseases used as input to the second step in the fuzzy logic system.

#### 3.2.1. Activities of Daily Living Impacted by Appearing Diseases

Once a deviation period is identified in the ADLs, we study in details the activities related to the behavior change period to discover the deviation cause(s). We, therefore, investigate the accuracy of the anomaly detection, per day and per hour of the day, in the behavior change period. Our goal here is to extract the activities that have deviated from the normal behavior and determine how the changes are evolving over time. Daily activities in this period are so mapped and compared with the normal life pattern to identify activities with a longer or shorter duration than expected. Missing activities are also identified meaning that the person skipped these activities during the day. Hence, our work belongs to *point anomaly* detection category (i.e, missing activity or activity with an unusually long/short duration) introduced in Section 2.3. For instance, anomalies in the activities of daily living, such as skip meals or sleep less, can be behavioral symptoms of chronic illnesses like diabetes, blood pressure, or mental illness, such as Alzheimer’s, depression, etc. In the rest of the article, the daily activities impacted by a disease will be called ADL-Disease (Activity Daily Living-Disease), where Disease stands for the suspected disease.

The list of ADLs-Disease can be established based on well-reviewed studies in the medical field or with the help of healthcare professionals.

The impact of the diseases on the ADLs can vary from one person to the other. Therefore, to assess the evolution of ADL-Disease, it is necessary to refer to the normal profile of each person built following the process described in Section 3.1.2.

For each ADL-Disease, we identify:

- The normal duration of the ADL-Disease corresponds to the minimum and the maximum durations of each ADL-Disease in the considered learning period. Both duration values are obtained after removing anomalies from the data used for profiling using clustering techniques as explained in Section 3.1.2. These two values will then be used at the fuzzification step (see Section 3.2.2);
- The normal frequency of ADL-Disease corresponds to the minimum and the maximum number of occurrences of each ADL-Disease per day in the considered learning period. Both frequency values are obtained after removing anomalies from the data used for profiling. These two values will then be used at the fuzzification step.

The ADLs-Disease introduced in this section are at the heart of the disease assessment phase. This step consists in identifying the possible disease(s) affecting the elderly person.

#### 3.2.2. Fuzzy Logic for Disease Level Assessment

Making recommendations in the healthcare domain is not an easy task due to the complexity of this domain and the critical effects on people’s life quality. Moreover, the disease level assessment is not a binary process where it would be easy to claim, according to several parameters and thresholds, that someone has or does not have a particular disease. Instead, our objective is to make recommendations about the probability to have one or more diseases with symbolic expressions, such as low, high, very high, etc., instead of the numeric values of the parameters. In this context, fuzzy logic has been identified as a substantial tool that is used to model human decision-making of an expert in a computer program such that the program can mimic the expert’s ability to solve problem [65]. The ability of fuzzy logic to handle imprecise and uncertain situations makes it the appropriate approach to realize medical diagnoses [66,67].

**Figure 2:**
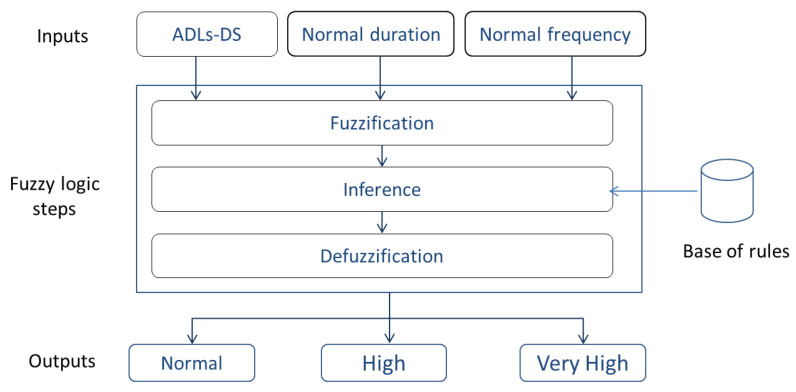
Fuzzy logic system for disease level assessment.

In the following, we explain how we exploit fuzzy logic to measure the disease level assessment for elderly people. Fuzzy in our context does not mean imprecise. On the contrary, if a data is not precisely known, it can be expressed using a confidence interval. Fuzzy logic consists of three main steps: (1) fuzzification, (2) inference, and (3) defuzzification. These three stages are integrated in our system, as depicted in Figure 2 and explained below.

1. **Fuzzification**: this first step in the fuzzy inference process involves a domain transformation where crisp inputs are transformed into fuzzy inputs. In other words, fuzzification is the process of transforming a real scalar value into a fuzzy value, also called linguistic value, in the interval [0, 1]. For this transformation, we apply a membership function as defined in (3).
  (3) $$\begin{array}{c} \mu_{(}x):X⟶\left[0,1\right]\\ x⟼\mu (x) \end{array}.$$
  There are many shapes of membership function like triangular, trapezoidal and convex. There are no precise rules on the choice of membership functions from three types defined in reference [64], but the most used are functions with the triangular and the trapezoidal shapes. In the linguistic representations, we use function with trapezoidal shape.
  In our system, the inputs are ADLs-Disease which represent linguistic variables. Each ADL-Disease is represented by three parameters:
  - **I**: the ADL-Disease identifier or label used in the activity pattern in Section 3.1.1.
  - **R**: the range of values for the duration and frequency of each ADL-Disease.
  - **F**: the fuzzy subset associated to the range of duration or frequency values. In our case, we define three fuzzy subsets: (1) Abnormal- when the ADL-Disease duration or frequency is less than the minimum duration in the normal behavior. (2) Normal when the ADL- Disease duration is between Min duration and Max duration in the normal behavior. (3) Abnormal+ when the ADL-Disease duration or frequency is greater than the maximum duration in the normal behavior.
  Our output is the disease level which depends on ADL-Disease, their fuzzy subsets and their number per day in the deviation period. The output consists on the following three subsets: (1) Normal, (2) High, and (3) Very high.
2. **Inference**: the second step in a fuzzy system constitutes the process by which fuzzy actions or operations are applied to input variables according to the rules defined in the system. In this step, we define the rules used for fuzzy reasoning also called approximate reasoning. In our case, the inference process will merge both input variables, namely ADL-Disease duration and the ADL-Disease frequency, to produce as output the disease level assessment. Several inference methods can be applied, the most popular being the use of the MIN/MAX operators [68].
3. **Defuzzification**: this last step in a fuzzy system converts a fuzzy value obtained from the inference step to a real value. Several methods can be used to perform this defuzzification. In our context, we use the method based on the center of gravity which is also the most used. For a resulting membership function $\mu_{R}\left(y\right)$, the center of gravity $y^{*}$ can be calculated by Equation (4). The result is a number between 0 and 1 which represents the disease level.
  (4) $$y^{*}=\frac{\int_{0}^{1}y\mu_{R}\left(y\right)dy}{\int_{0}^{1}\mu_{R}\left(y\right)dy}=\frac{\sum_{k=0}^{n}y_{i}\mu_{yi}}{\sum_{k=0}^{n}\mu_{yi}}.\;$$

In this section, we described a solution to monitor the behavior of elderly people and notify her/his caregivers about a possible appearing disease. In the following section, we propose an experimental evaluation of our proposal using a real dataset.

## 4. Use Case

In this section, we propose an evaluation of our different proposals using a real dataset. We first present the dataset corresponding to our use case. We then detail the generation of the normal behavior pattern. By plotting the daily score along time, we exhibit the period when the score changes over time and then identify the abnormal daily activities observed during the period of behavior change. Finally, using the activities anomalies, we illustrate the proposed decision support system to identify a suspected disease level.

### 4.1. Dataset

The dataset used for our analysis is provided by Washington State University’s CASAS program (http://casas.wsu.edu/) [14]. CASAS (Center for Advanced Studies in Adaptive Systems) aims to provide aid to residents using smart home technology. Real-time data is, therefore, collected from sensors to analyze and monitor residents’ health and behavior to improve future smart home living.

**Figure 3:**
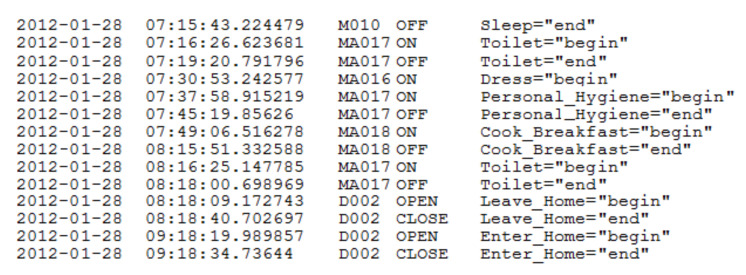
Activity-labeled smart home sensor data.

During our experimentations, we used one of the public datasets (named HH120) [14] which was also used in other works like [16]. This dataset contains sensor data that was collected in the (smart) home of a volunteer adult. It includes one unique subject, covering a total of 63 days. This smart home is equipped with passive infrared (PIR) motion sensors and magnetic door sensors (25 sensors). All the data used in this article was handled in an anonymized way. In addition to the date, time, sensor localization, and a sensor message, each reading is assigned an activity label. The activity labels are provided by a real-time activity recognition algorithm called AR [14]. AR is trained from human-annotated ground truth data to label sensor events using the following set of labels: {Cook, Eat, Sleep, Personal hygiene, Leave home, Enter home, Bathe, Relax, Bed-toilet transition, Wash dishes, Other activity}. Figure 3 provides a sample of collected and labeled smart home sensor data. In this figure, we consider:

- “M”: infrared motion sensors,
- “MA”: wide-area infrared motion sensors, and
- “D”: magnetic door sensors.

From this set of activities labeled, we are in our study interested in: sleeping, eating meals, taking a shower, leaving home, and going to toilet. For each activity, we use a start time which corresponds to the time when the activity state is equal to begin. Moreover, we use the duration of the activity which is equal to the difference between start time (activity = “begin”) and end time (activity = “end”). The datasets do not provide any medical information. For building the normal behavior model, we selected the first month as a data used for profiling, while the rest of the available data is used to test the effectiveness of our proposals.

### 4.2. Learning for Building the Normal Behavior Pattern

**Figure 4:**
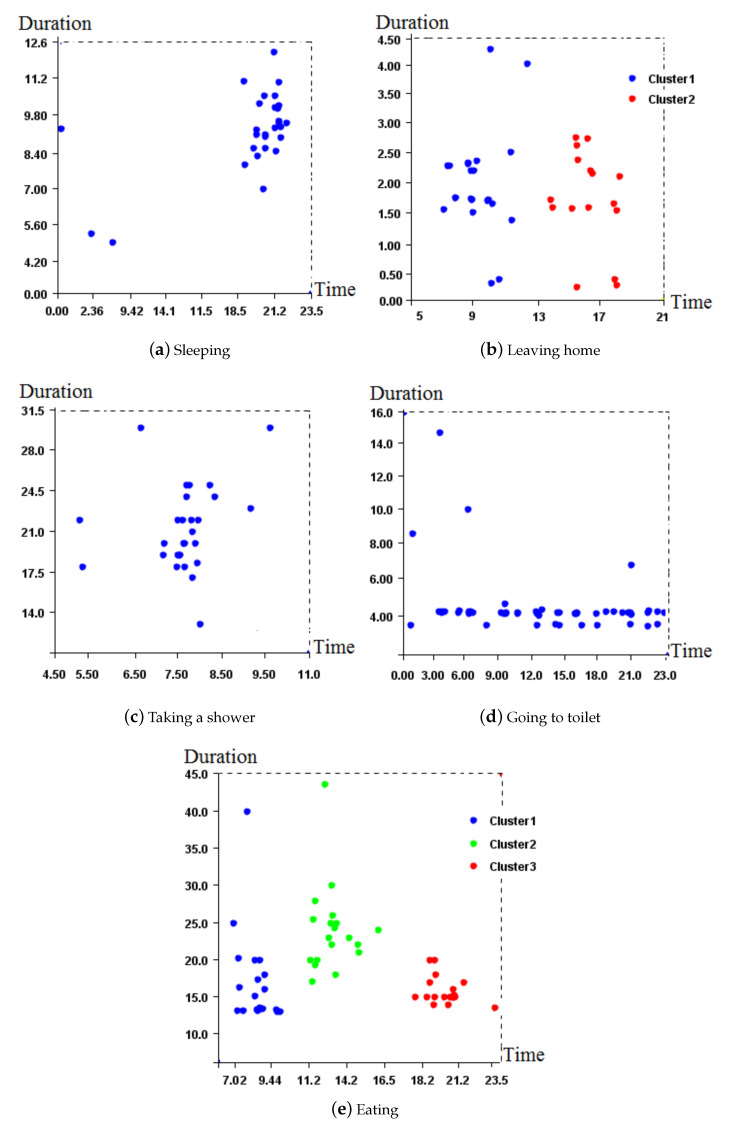
DBSCAN clustering for detecting anomalies.

For building the normal behavior pattern from the first month, we follow the different learning steps presented in Section 3.1.2. We apply the DBSCAN algorithm on the first month of dataset for clustering. Then, we eliminate activities out of the identified clusters and marked as noise. DBSCAN has two parameters; one is *min_pts* which is the minimum number of points in a cluster, and the other is *Eps* which is the maximum distance between two data points for them to be considered in the same cluster. While learning, data out of *Eps* would be considered out of clusters and so marked anomalous. Based on the heuristic proposed in reference [63], we set *min_pts* to 4 and *Eps* to 2. Results obtained for each activity are depicted in Figure 4.

Figure 4e illustrates 3 clusters, which represent the 3 daily meals. The elderly person used to have her breakfast at around 9:44 a.m. for a maximum of 25 min. Figure 4e shows that this person once had her/his breakfast for 40 min, which is abnormal compared to the usual behavior.

**Figure 5:**
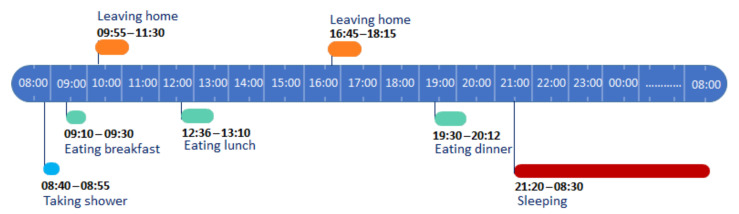
Normal behavior pattern.

We then eliminate point anomalies and calculate, for every activity in the data used for profiling representing one month of collected data, the average start time, and average duration. Figure 5 illustrates the daily behavioral model thus generated using the previous learning step.

### 4.3. Behavior Change Period and Anomalous Activities

**Figure 6:**
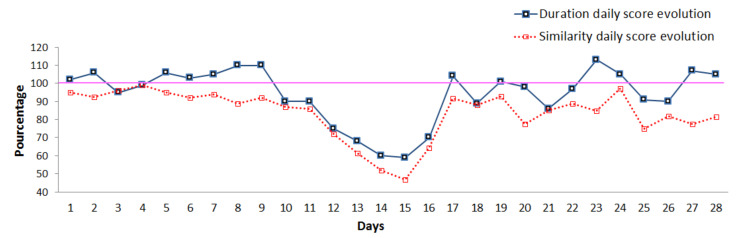
Daily scores evolution.

In the first stage of our experiments, we computed the daily scores introduced in Section 3.1.3. By plotting scores, we can observe the day by day evolution of the elderly person’s behavior. Thus, it is possible to identify trends in the daily evolution scores, as shown in Figure 6, where we observe a decrease compared to the previous routine activity.

**Figure 7:**
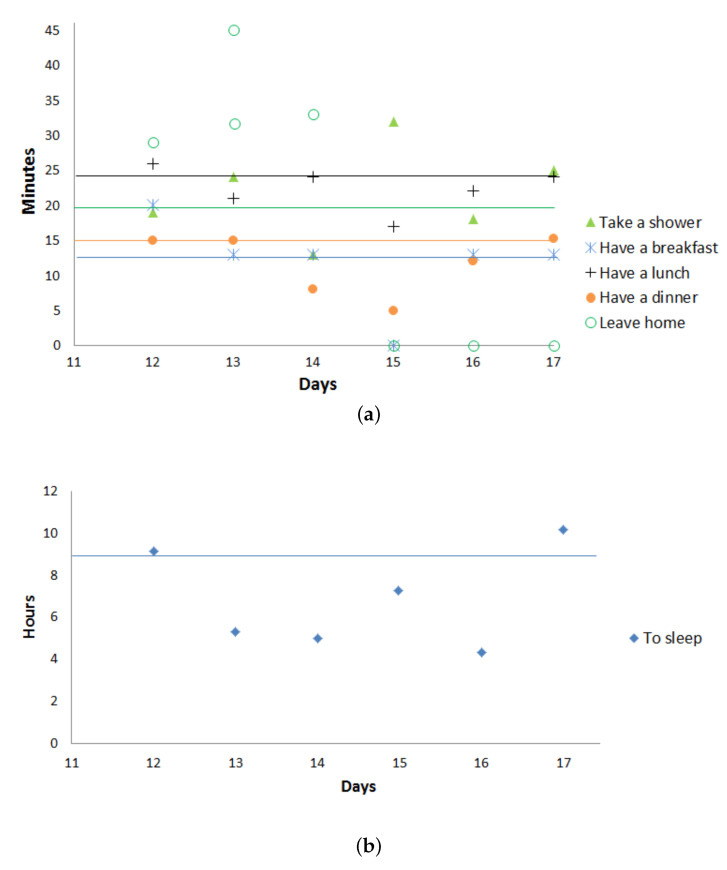
(**a**) Activities durations in observed days; (**b**) sleeping duration.

**Figure 8:**
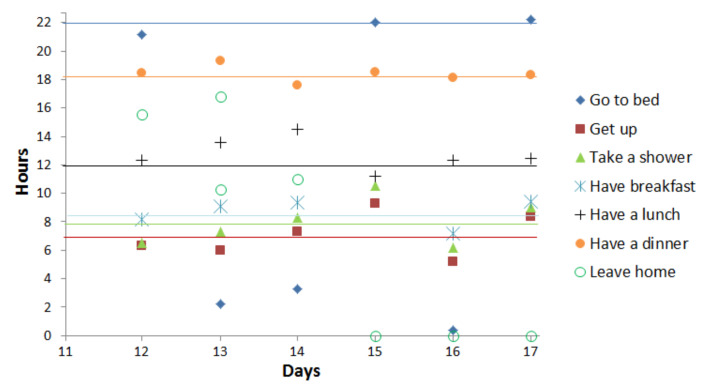
Activities start time in observed days.

In the second stage of our experiments, we focus on the deviation period (days with decreasing/increasing scores) to detect point anomalies due to a missing activity or activities with unusually long or short durations. Therefore, we plot in Figure 7 and Figure 8 the duration and start time for 3 activities (sleeping, eating (breakfast, lunch, dinner), and taking a shower). In these figures, the average start time and the average duration in the normal behavior pattern are represented for each activity by an horizontal line.

At days 13 and 14, Figure 7 and Figure 8 reveal unusual sleep times, shorter than usual, as well as later times to go to bed (2:00 a.m. and 4:00 a.m.). The results also indicate that day 15 is a day with unusual activity because the elderly skipped a lunch. At the same day, the elderly performs in more times than usual the activity taking a shower and sleeping. We notice, more precisely at days 15, 16, and 17, a reduction in the duration of leaving home that can reach 0 on these days, which explains that the person does not leave the home. During these 6 days, we detect 2 types of point anomaly due to missing activity and activities with unusually long/short durations.

As mentioned previously, the activity going to toilet that occurs several times a day is treated separately. For this activity, we focus on:

- The frequency per 2-h slots during the day.
- The Max duration per day.

**Figure 9:**
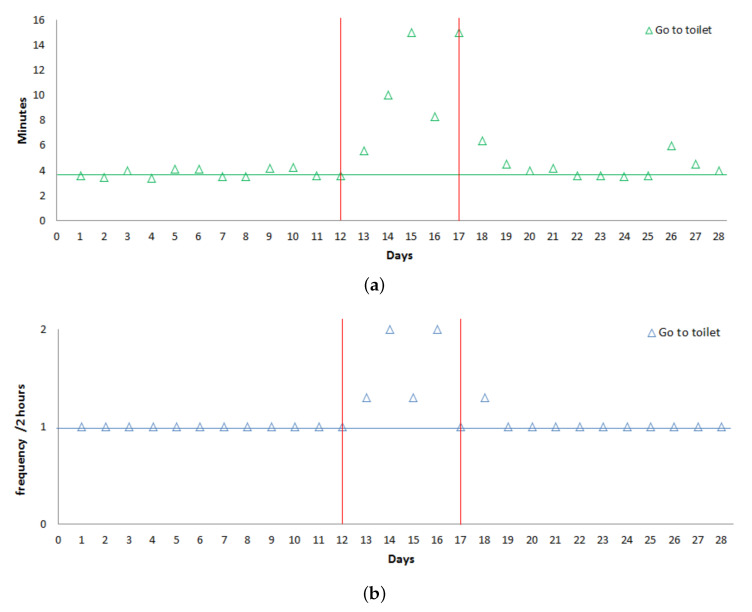
(**a**) Frequency go to toilet. (**b**) Duration go to toilet.

Figure 9 shows that for the activity going to toilet both these parameters increase in the deviation period compared to the normal behavior (represented with the horizontal line).

All these anomalies may be a signal of sickness (urinary tract infection, gastrointestinal problem, etc.), so our system may send an alert message to inform remote caregivers.

### 4.4. Illustration of the Decision Support System

The objective of our decision support system is to notify family members and/or caregivers about suspected diseases detected using behavior changes. In the following, we illustrate how this process works and how suspected diseases can be identified. Therefore, we consider the identification of a gastrointestinal problem for the elderly person. Even if our objective is to detect diseases in which symptoms are not as obvious as the ones of gastroenteritis, this pathology was easily identifiable for the elderly observed in our dataset. We so decided to exploit this disease to explain how our decision support system works.

As we explained in Section 3.2, our decision support system follows a two-step process.

The first one consists in identifying the ADLs impacted by the gastrointestinal problem, called ADL-Gastrointestinal in the following and the fuzzy rules used for decision-making. Based on studies recognized in the medical world [69,70,71], a list of ADLs impacted by a gastrointestinal disease can be established, as illustrated in Table 1. For each ADL-Gastrointestinal, we then identify the maximum and the minimum normal duration (see Table 2), and the maximum and the minimum normal frequency (see Table 3). These values are extracted from the normal behavior introduced in Section 4.2. They are used here to define the range of values where the activities should be considered as normal or abnormal, as detailed in Section 3.2.2. For example considering the activity “Sleeping” in the elderly person’s normal behavior, s/he can sleep between 31,200 and 40,800 s per day. If the duration of sleeping is less than 31,200 s, we will consider the change as an abnormal state and more precisely as abnormal– since the duration is less than the normal minimum threshold. As the same manner, if the duration is more than 40,800 s, we will consider the state as an abnormal+. These ranges will be used at the fuzzification step of our solution.

In our example, the gastrointestinal illness is discovered according to the abnormality in ADLs-gastrointestinal, the ADLs that can be impacted in case of gastrointestinal problems. Moreover, the gravity of this gastrointestinal problem will be proportional to the number of anomalies observed in the ADLs-gastrointestinal along the deviation period. In our example, we observe 8 abnormal states related to the duration and frequency of ADLs-gastrointestinal and define three illness levels: Low, High, and Very high, with fuzzy rules used for decision-making as follows:

- If less than 2 changes in the ADLs-Gastrointestinal are observed, then the level of the gastrointestinal problem is low.
- If a change in the ADLs-Gastrointestinal with at least 2 or 3 abnormal states is observed every day in the deviation period, then the level of the gastrointestinal problem is considered as high.
- Finally, if a change in the ADLs-Gastrointestinal with at least 4 abnormal states is observed every day during the deviation period, the level of gastrointestinal problem is supposed very high.

Of course, in a real application, the thresholds defining the illness levels should be defined with the help of an healthcare expert. In the following, we give some examples of fuzzy rules, used for decision-making, to illustrate the different levels introduced previously:

Rule 1: If eating duration is Normal and eating frequency is Normal and sleeping duration is Normal and sleeping frequency is Normal and leaving home duration is Normal and leaving home frequency is Normal and going to toilet duration is Normal and going to toilet frequency is Normal, then gastrointestinal level is Low.

Rule 2: If eating frequency is Abnormal- and leaving home frequency is Abnormal- and sleeping duration is Abnormal-, then gastrointestinal level is high.

Rule 3: If eating frequency is Abnormal- or eating duration is Abnormal- and leaving home frequency is Abnormal- or leaving home duration is Abnormal- and going to toilet duration is Abnormal+ or going to toilet frequency is Abnormal+ and sleeping duration is Abnormal- or sleeping frequency is Abnormal-, then gastrointestinal level is Very high.

**Figure 10:**
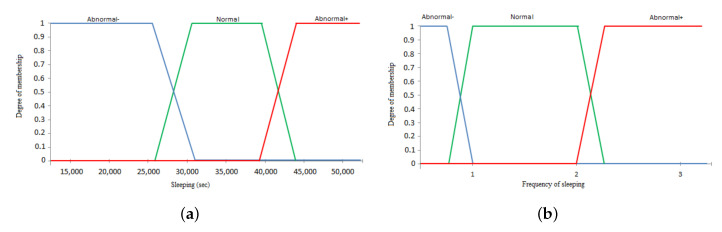
The input ADL-Gastrointestinal: Sleeping.

**Figure 11:**
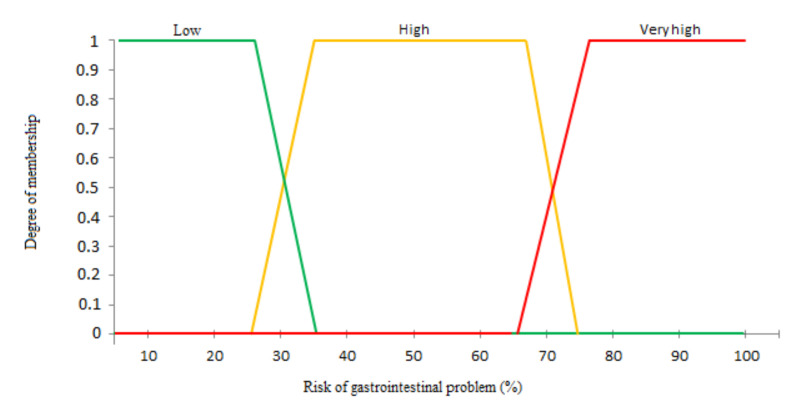
Output of decision support system.

**Figure 12:**
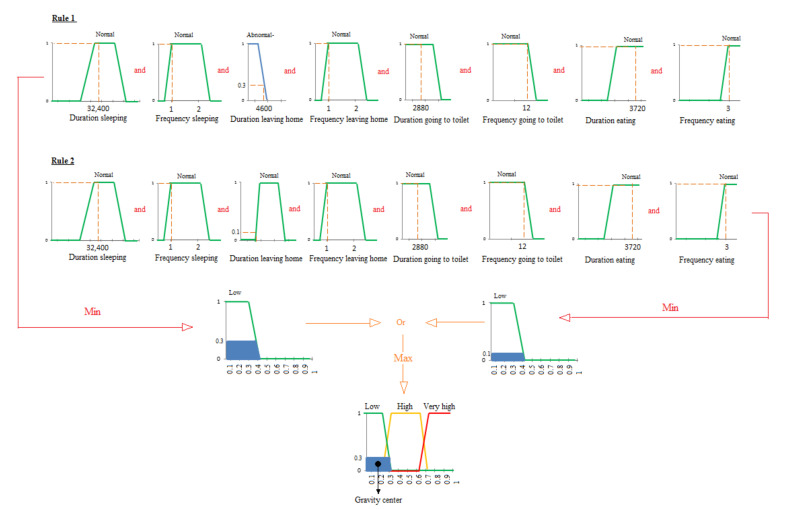
Inference example by the Mamdani method applied to day 12 in the deviation period.

At the second step of our decision support system, fuzzy logic is used to measure the disease level assessment for elderly people. As explained in Section 3.2.2, the fuzzy logic process consists of three main steps applied to our case study as follows:

1. **Fuzzification**: at this step, the duration and frequency of the ADLs-Gastrointestinal defined in Table 2 and Table 3 are used as input to the system. As explained in Section 3.2.2, a membership function with a trapezoidal shape is applied to transform a real scalar value into a fuzzy value, also called linguistic value. Thus, the real values of the the duration and frequency for the ADLs-Gastrointestinal are transformed into linguistic values which are Abnormal-, Normal and Abnormal+. The example of input is shown in Figure 10. In this example, the ranges of values for the duration (Figure 10a) and frequency (Figure 10b) of one ADLs-Gastrointestinal are represented: sleeping with its fuzzy subsets. Note that the projection of the value of the x axis on the y axis provides the fuzzy value. For example, in Figure 10a, the fuzzy values of the sleeping duration 3000 s corresponds to 0.2 Abnormal- and 0.6 Normal. At the fuzzification step, we use the trapezoidal shape to plot the three subsets: Low, High, and Very high, of the output of our decision support system, as depicted in Figure 11.
2. **Inference**: at this step, fuzzy actions or operations are applied to input variables according to the fuzzy rules previously defined. In our case, the inference will merge the input variables related to duration and frequency of the four ADLs-Gastrointestinal to find in the output the gastrointestinal disease level. As explained in Section 3.2.2, the max-min method, also known as the Mamdani method, is used to activate the rules and calculate the implication. An example of this method is cited in Figure 12, which is applied to the first day of the deviation period with values of ADLs-Gastrointestinal, given in Table 4.
  At the first day of the deviation period, we observe one ADL-Gastrointestinal with an abnormal value which is the duration of leaving home, so the level of the disease in the output is in the first illness level, Low, since we have one ADL-Gastrointestinal with an abnormal value as defined for outputs in the first step of decision-making process. Figure 12 shows that the fuzzy values are equal to 1 for all ADLs-Gastrointestinal except for the duration of leaving home in which projection gives two values: the first value is in the Abnormal- range, and the second value is in the Normal range. In this case, we apply two fuzzy rules given in Figure 12. With the first rule and the Abnormal- duration of the activity leaving home, the 4600 s of this duration gives us 0.3 as fuzzy value. So, according to the Mamdani principle [68], we use the MIN operator since we have the operator AND between the 8 conditions, so we select the minimum between 0.3 and 1. We do the same operation for the second rule, which gives us the fuzzy value 0.1 for the duration of leaving home. The last step consists in the aggregation between both rules performed using the MAX operator between the both fuzzy values.
3. **Defuzzification**: at this step, the level of the gastrointestinal problem is estimated using a number between 0 and 1. Therefore, the method based on the center of gravity explained in Section 3.2.2 is used since it is the most popular in the literature. This principle is illustrated in the last part of Figure 12 by calculating a center of gravity of the resulting fuzzy output subset(s). In our example, the illness level provided by our system for the first day in the deviation period is 0.19.
  As shown in Table 5, the degree of the disease is calculated per day in the deviation period. This means that a daily decision can be made.
  Based on the level of the gastrointestinal problem, the system also determines the type of alert that is sent to the caregiver for her/him to make an appropriate decision. Our system, therefore, integrates three different types of alert:
  - Minor gastrointestinal problem.
  - Important gastrointestinal problem.
  - Major gastrointestinal problem.
  In the example described in Table 5, the gastrointestinal problem evolves quickly and the caregiver should intervene as soon as possible according to the health state of the elderly person. For an elderly person suffering from kidney disease, the caregivers must intervene as soon as s/he receives the first alert about a “minor gastrointestinal problem”.

## 5. Evaluation of the Proposed Method

In this section, we propose an experimental study to validate our approach. Our objective with these experiments is to highlight the effectiveness and to assess the performance of our system for detecting elderly’s behavior changes over time. Therefore, we compare the results obtained for our framework with those provided by the solution proposed by Caroux et al. and described in reference [61].

As in our work, Caroux et al. introduced a score to evaluate how strictly an activity matches with the user’s routine. This activity score is between 0 and 1; 0 means that the activity has not been performed, according to the user’s routine, whereas 1 indicates that the sensed measures strictly match the user’s routine. The behavior routine is provided by the elderly person themselves or by another adult. The outcome is a pattern of daily activities (e.g., breakfast takes place between 7:00 a.m. and 9:00 a.m.). As explained in Section 2.4, the approach proposed by Caroux et al. [61] does not consider ADLs, like toileting and leaving home. It is applied to five ADLs: meals preparation, go to bed, get up, taking a shower, and getting dressed. The score of each activity is computed using a specific formula. The daily score is then calculated as the average of activity scores. To compare the effectiveness of both scores for detecting behavior changes, we computed the daily scores for both approaches using the same database (HH120) [14] for all common activities (meals preparation, go to bed, get up, and taking a shower).

**Figure 13:**
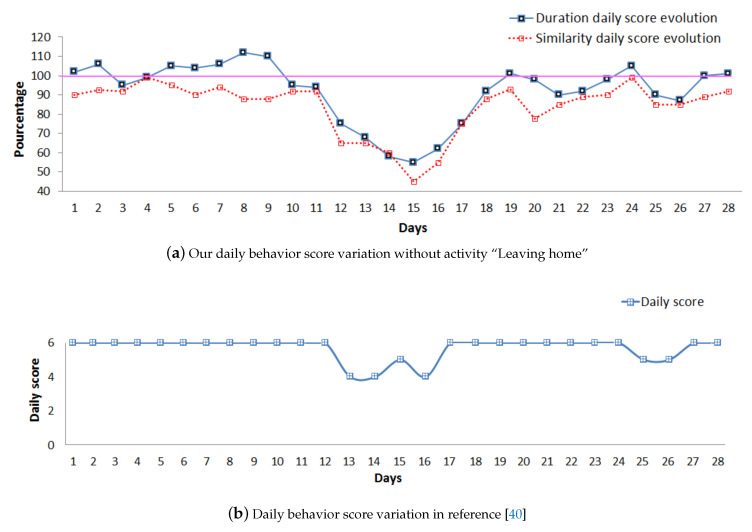
Daily scores for (**a**) our approach and (**b**) Reference [61]’s approach.

By plotting the evolution of daily scores along time in Figure 13, we can observe the day-by-day evolution of the elderly person’s behavior for both approaches. With our approach, as explained in Section 4.3, it is possible to identify trends in the daily evolution scores. In Figure 13a, we thus observe that both the duration and similarity scores begin to deviate from the normal behavior (daily scores equal to 100%) starting from day 7. Our duration score increases, and the similarity score decreases, during the 3 first days. Both scores then start decreasing gradually until day 15. The daily score proposed by Caroux et al. [61] illustrated in Figure 13b also shows a deviation from the normal behavior. Contrary to our results, this deviation period is suddenly detected starting from day 13 (score = 6 at day 12 to score = 4 at day 13). Furthermore, the approach proposed by Caroux et al. does not detect any change from the normal behavior from day 8 to day 12.

**Figure 14:**
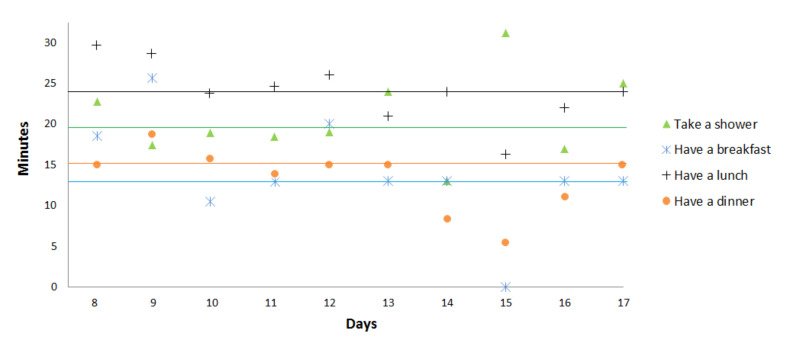
Duration of activities for the observed days.

In the second stage of our experiment, we study the deviation period separately to prove that the behavior of the elderly person is gradually changing during the 5 days (from day 8 to day 12). Therefore, we focus on the activities to detect point anomalies. Therefore, we start by plotting in Figure 14 the duration of the activities in the target deviation period. At days 8 and 9, Figure 14 reveals unusual lunch and breakfast times, longer than usual. At day 8, the elderly person takes more time than usual for his/her activity taking shower. These two point anomalies related to the duration of the activities are well detected by our duration score (score exceeds 100% for both these days). On the contrary, they are not detected considering the score proposed by Caroux et al. [61] since this score does not take into account the duration of the activities. Their score comprises two temporal dimensions which are (1) the time of the day at which the activity occurs and (2) the minimal duration over which the activity is supposed to be performed. The second temporal dimension (minimal duration) is considered only for activity taking a shower score.

The problem of duration anomalies detection also appears at day 15. Our daily scores, presented in Figure 13a, reach their minimum values (similarity = 45.32% and duration = 58.47% ), while the daily score proposed by Caroux et al. [61], shown in Figure 13b, is still high. Figure 14 reveals that at day 15 the elderly person did not take breakfast and performed less times than usual the activities eating lunch and eating dinner. Moreover, more time than usual was taken for the activity taking a shower. These three duration anomalies are not detected by the daily score proposed by Caroux et al. [61], and the increase of this score at day 15 is due to missing activity eating breakfast.

The obtained results confirm the effectiveness of our proposed score in detecting behavior changes over time. Our approach is indeed more sensitive for detecting the gradual changes in the elderly behavior over time. This is due to the model of normal behavior built on the basis of the past activities of the elderly person which are more precise and complete than that given by the elderly person themselves. In addition, our score is more sensitive since it covers more activities and takes into account the duration of the activities. This allows us to follow if the elderly person starts to take more/less time to produce their activities.

In the following, we evaluate the overall performance of our solution using two different metrics: Sensitivity (S) and Accuracy (A). As explained in Reference [72], Sensitivity is used to measure the fraction of positive patterns (Tp: True positive and Fn: False negative ) that are correctly classified (perfect Sensitivity score is equal to 1). Accuracy corresponds to the ratio of correct predictions (Tp: True positive and Tn: True negative) over the total number of instances evaluated (N = 28 days in our case). Table 6 shows the scores obtained for the Sensitivity and Accuracy indicators for both solution. In this table, we consider:

- Tp: the number of days correctly identified as abnormal days (i.e., with at least one abnormal activity).
- Tn: number of days correctly identified as normal days (i.e., with no abnormal activity).
- Fn: number of days incorrectly identified as normal days.

We can observe that the scores obtained with our solution for both Sensitivity and Accuracy metrics outperform those computed for the solution proposed by Caroux et al. This confirms that our approach better identifies normal and abnormal days.

## 6. Discussion and Conclusions

In this article, we presented a framework to detect behavior changes in the elderly person’s usual behavior. Our framework relies on the construction of a behavioral model integrating several previously identified daily activities. Thanks to this model, changes in the elderly person’s behavior can be detected, and not only sudden changes, such as a fall or a temporary illness, but also silent changes over a long period of time. Our framework also exploits these changes detected in the usual behavior to discover the possible diseases affecting the elderly. Therefore, a decision-support system has been proposed. Our decision support system helps caregivers to discover and identify the (level of the) disease. They can then better react accordingly, by delivering drugs or deploying dedicated assistance to the patient. In the future, we plan to integrate contextual elements, such as weather conditions and other information on health conditions, to refine our detection of behavioral changes, since these parameters may also have an impact on the usual behavior. Although elderly people have fairly regular lifestyles and rhythms of life that usually do not change much from one to day to the other, unlike other types of people, it will be important in our future work to adapt our work for the elderly person who does not fit into this regularity rule. Moreover, since the profile of elderly people may be very heterogeneous in terms of autonomy, illness, dependence, age, etc., we aimed to improve the decision-making process according to the person’s health status.

## Figures and Tables

**Table 1:** Activities of Daily Living related to Gastrointestinal problem symptoms (activities of daily living (ADL)-Gastrointestinal).

Activities	Actions
Decrease in leisure activities	Decreasing of leaving home
Go to toilet disorder	Increasing frequency of going to the toilet
	Increasing the time spent in the toilet
Eat disorder	Eating too little
Sleep disorder	Sleeping too much or too little

**Table 2:** Average duration per day of the ADL-Gastrointestinal.

Activities	Leaving Home	Going to Toilet	Eating	Sleeping
Min (sec)	9000	2200	2880	31,200
Max (sec)	14,400	3600	4500	40,800

**Table 3:** Normal frequency of the ADL-Gastrointestinal in a day.

Activities	Leaving Home	Going to Toilet	Eating	Sleeping
Min	1	10	3	1
Max	2	12	4	2

**Table 4:** Duration and frequency of ADLs-Gastrointestinal in the first day of the deviation period.

Activities	Leaving Home	Going to Toilet	Eating	Sleeping
Duration (sec)	4600	2880	3720	32,400
Frequency	2	12	3	2

**Table 5:** The level of a gastrointestinal problem in deviation period.

Day in Deviation Period	11	12	13	14	15	16	17	18
Gastrointestinal problem level	0.19	0.38	0.55	0.7	0.74	0.75	0.51	0.3
	low	high	high	very high	very high	very high	high	low

**Table 6:** Metrics for evaluating performance of our proposed method compared with reference [61].

	Tp	Tn	Fn	Sensitivity	Accuracy
				$\frac{\mathrm{Tp}}{\left(\mathrm{Tp}+\mathrm{Fn}\right)}$	$\frac{\mathrm{Tp}+\mathrm{Tn}}{N}$
Our approach	11	16	2	0.85	0.96
Caroux et al.	6	16	5	0.54	0.78

## Abbreviations

The following abbreviations are used in this manuscript:

USEFIL	Unobtrusive Smart Environments For Independent Living
CASAS	Center for Advanced Studies in Adaptive Systems
ADL	Activity Daily Living
BADL	Basic Activity Daily Living
IADL	Instrumental Activity Daily Living
ADL-Disease	Activity Daily Living-Disease
ADL-Gastrointestinal	Activity Daily Living-Gastrointestinal
